# Punitive preferences, monetary incentives and tacit coordination in the punishment of defectors promote cooperation in humans

**DOI:** 10.1038/srep10321

**Published:** 2015-05-19

**Authors:** Andreas Diekmann, Wojtek Przepiorka

**Affiliations:** 1 Chair of Sociology, ETH Zurich, Clausiusstrasse 50, CLU D 3, CH-8092 Zurich, Switzerland; 2 Department of Sociology, University of Oxford, Manor Road, Oxford, OX1 3UQ, UK; 3 Department of Sociology, Utrecht University, Padualaan 14, Utrecht, 3584 CH, The Netherlands

## Abstract

Peer-punishment is effective in promoting cooperation, but the costs associated with punishing defectors often exceed the benefits for the group. It has been argued that centralized punishment institutions can overcome the detrimental effects of peer-punishment. However, this argument presupposes the existence of a legitimate authority and leaves an unresolved gap in the transition from peer-punishment to centralized punishment. Here we show that the origins of centralized punishment could lie in individuals’ distinct ability to punish defectors. In our laboratory experiment, we vary the structure of the punishment situation to disentangle the effects of punitive preferences, monetary incentives, and individual punishment costs on the punishment of defectors. We find that actors tacitly coordinate on the strongest group member to punish defectors, even if the strongest individual incurs a net loss from punishment. Such coordination leads to a more effective and more efficient provision of a cooperative environment than we observe in groups of all equals. Our results show that even an arbitrary assignment of an individual to a focal position in the social hierarchy can trigger the endogenous emergence of more centralized forms of punishment.

The second-order free rider problem is a key problem in social theory^1^,^2^,^3^,^4^. Actors cooperate at high rates if defectors can expect to be punished. If punishment is voluntary and costly, self-regarding actors will refrain from punishing defectors and first-order cooperation will break down or not emerge at all. The punishment of defectors can thus be regarded as a second-order public good dilemma. It has been shown that the provision of a peer-punishment mechanism can sustain first-order cooperation if a certain proportion of group members punish defectors at a cost to themselves^5^,^6^,^7^. The punishment mechanism employed in these studies can be conceptualized by a (second-order) linear public good game. It has been argued that punitive preferences are a key driver of peer punishment^8^,^9^,^10^. However, it is an open question how punitive preferences could have evolved in humans, as peer punishment does not always lead to a net benefit for the punisher and the group^11^,^12^,^13^,^14^.

In many situations in which defection can be punished, only one group member’s punishment is necessary and sufficient to establish cooperation. For example, in a group of people who are affected by the norm violation of another person (e.g., a neighbor playing too loud music), only one person’s intervention may be necessary and sufficient to stop the transgressor and benefit the group. In this case, the punishment mechanism can be better conceptualized by a (second-order) step-level public good game^15^. Moreover, if the benefits outweigh the punishment costs, it may be preferable even for self-regarding actors to engage in the punishment of defectors^16^. It has been shown theoretically that conceptualizing the second-order public good by a nonlinear production function allows for the coexistence of punishers and non-punishers in large groups of unrelated individuals^17^,^18^.

With a certain amount of punishment sufficing to establish cooperation, a coordination problem can arise with regard to which group members should exercise how much punishment on the defectors^19^. Too little punishment may fail to establish cooperation; too much punishment may succeed but cancel or even exceed the benefits. Appointing a designated punisher^20^,^21^,^22^, or establishing a pool punishment institution^23^,^24^,^25^,^26^,^27^ can solve this coordination problem. However, both mechanisms presuppose the existence of a central authority that consolidates and legitimizes the use of violence. How such an authority emerges in the first place remains in need of an explanation.

In accordance with a conceptual framework of institutional emergence^28^ and insights from game theoretic analyses^29^,^30^, we argue that individuals’ distinct ability to punish defectors may be key to explain the transition from peer-punishment to more centralized forms of punishment. In particular, we argue that in a state devoid of any norm enforcing institutions, social order will be maintained by the “strongest” individuals, who emerge as the “violence specialists”^28^ in a group. This idea is consistent with results from simulation experiments investigating the evolution of cooperation in the spatial prisoner’s dilemma game. These simulations show that diversity in agents’ abilities to translate their prisoner’s dilemma payoffs into fitness scores, promotes the evolution of cooperative clusters led by high-ability agents^31^. Moreover, evidence from laboratory experiments with step-level public good games suggests that an unequal distribution of punishment costs across group members may tacitly single out the strongest member to carry out the punishment efficiently^32^. In linear (second-order) public good games, the coordinating effect of punishment cost heterogeneity seems harder to achieve without communication^33^,^34^,^35^.

We conduct a laboratory experiment to investigate how the interplay of punitive preferences, monetary incentives and actors’ relative strength affects the punishment of defectors and hence first-order cooperation. We employ a punishment mechanism in which only one group member is necessary and sufficient to produce the second-order public good. We vary the structure of the punishment situation (i.e. second-order public good game) while keeping the complexity of the first-order cooperation problem at a minimum. Our theoretical argument is based on a game theoretic model, which allows us to derive clear hypotheses that can be put to an empirical test.

In a group of four equally endowed individuals, one randomly chosen group member has the opportunity to “steal” half the endowment of the other three. While not stealing maintains the status quo of equal benefits for all, by stealing, one group member makes a gain at the expense of the other three. Thus, the first-order cooperation problem is comparable to a common-pool resource dilemma in which three group members do not over extract the resource while a fourth group member has the opportunity to over extract. In case of theft, the three group members can decide independently whether to reclaim the stolen endowment *U*_*i*_. If at least one of them decides to reclaim it, the initial endowments will be restored for all group members (including the thief). However, every group member who decides to reclaim the endowment incurs a cost *K*_*i*_, and if no one decides to reclaim the endowment, the thief keeps the entire “loot” of 3*U*_*i*_.

Our experiment comprises 30 rounds and after each round, the groups of four are disbanded and randomly formed anew. In the first 15 rounds (part 1), reclaiming the stolen endowment does not impose a penalty on the thief. As from round 16 (part 2), if at least one group member reclaims the stolen endowment, initial endowments are restored and the thief incurs a penalty *P*; the size of the penalty is independent of the number of other group members’ decisions to also reclaim the money. Thus, only in the second part does reclaiming correspond to the standard notion of punishment, where both the punisher and the punished incur a cost. To keep the language simple, we will call a person’s decision to reclaim the stolen endowment in both parts “punishment”, and a person’s decision to steal or not to steal part of others’ endowments “defection” and “cooperation”, respectively.

Based on the structures of the second-order public good games devised in Table 1 and the game theoretic model predictions devised in the Methods section, we can derive testable hypotheses. In one variant of the second-order public good game – the missing hero dilemma (MHD) – the punishment costs exceed the benefits (*K*_*i*_ > *U*_*i*_ > 0) and self-regarding actors will not punish^36^. In another variant of the game – the volunteer’s dilemma (VOD) – the benefits exceed the punishment costs (*U*_*i*_ > *K*_*i*_ > 0) and self-regarding actors will exert punishment with a certain probability^37^. So, if we assume all actors to be self-regarding, we can expect punishment rates to be zero in the MHD (hypothesis H1) and hence to be higher in the VOD than in the MHD (H2).

With both the MHD and the VOD we employ the symmetric game, in which all group members have the same punishment costs (*K*_*i*_ = *K*_*j*_ " *i* ≠ *j*), and an asymmetric game in which one “strong” group member has slightly lower punishment costs than the rest of the group (*K*_*i*_ < *K*_*j*_ " *j* ≠ *i*)^38^. Assuming self-regarding actors, this distinction will not make a difference in the MHD (see H1). In the symmetric VOD, coordination on only one group member carrying out the punishment is hardly possible without communication. However, in the asymmetric VOD, we expect groups to be able to tacitly agree on mainly the strong person to punish defectors (H3)^29^. Consequently, we expect defectors to be punished at a higher rate (H4) and more often by one person only (H5) in the asymmetric than in the symmetric VOD. Hence, when a penalty for punished defectors is introduced in the second half of the experiment, we expect (first-order) defection rates to be lower in the asymmetric VOD than in the other conditions (H6).

If we assume also other-regarding actors with punitive preferences, we can expect the punishment rate in the MHD to be larger than zero (H1*a*). Moreover, since we would expect monetary incentives to be stronger than or even complementary to punitive preferences in their driving punishment decisions, assuming also other-regarding actors does not affect hypothesis H2, and hypotheses H3 through H5 can also be tested with the MHD. Finally, we can extend hypothesis H6 to reflect the empirical punishment rates. Correspondingly, (first-order) defection rates should be negatively correlated with the rates at which defectors are punished. In particular, defection will be lower in the asymmetric VOD than in the symmetric MHD (H6*e*).

## Results

**Figure 1:**
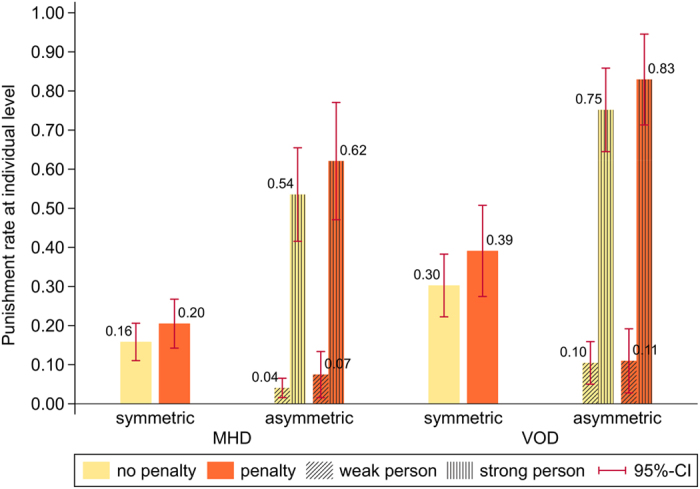
The figure shows the individual punishment rates across experimental conditions and person types. The significant rates in the symmetric MHD condition confirm that punitive preferences partly drive punishment decisions. The fact that the rates are significantly higher in the VOD than in the MHD conditions indicates that monetary incentives also matter. In both asymmetric conditions, the strong group member is much more likely to punish defectors than a weak group member. This shows that groups are able to tacitly coordinate on mainly the strong group member to punish defectors based on differences in punishment costs alone. See section S2 in the *SI* for further details on the data analysis.

Figure 1 shows the punishment rates at the individual level across experimental conditions and person types. Note first that punishment rates within experimental conditions and person types do not substantially depend on whether a penalty is imposed on a punished defector. Although this result is interesting in itself, because it suggests that subjects are not primarily driven by spite in their punishment decisions^39^, we will not further expand on it here (see the Methods section for a brief discussion). If not otherwise stated, we will base subsequent analyses regarding the punishment of defectors on the pooled data from both parts of the experiment. Section S2 in the Supplementary Information (*SI*) contains the separate analyses.

It becomes immediately apparent that our results support hypothesis H1*a* rather than H1. There is a significant proportion of punishment in the MHD, which can be attributed to group members with punitive preferences punishing defectors. The relation between punishment costs and benefits also matters. In line with hypothesis H2, punishment rates are significantly higher in the VOD than in the MHD, overall (χ^2^_(1)_ = 15.61, *p* < 0.001) as well as in the symmetric (χ^2^_(1)_ = 9.04, *p* = 0.003) and asymmetric games (χ^2^_(1)_ = 9.30, *p* = 0.002) separately. Moreover, for the asymmetric VOD and MHD, Fig. 1 shows that the strong group member is overwhelmingly more likely to carry out the punishment than the other group members^40^. These results clearly support our hypothesis H3 and prompt the next two important questions. Does the possibility to tacitly agree on the strong member to punish defectors in the asymmetric games indeed lead to higher rates at which defectors are punished than in the symmetric games? And, is punishment in the asymmetric games more efficient because carried out more often by one person only than in the symmetric games?

In line with H4, the rates at which defectors are punished, that is the rates at which *at least one* group member punishes a defector, are higher in the asymmetric MHD and VOD (60% and 81%, respectively) than in the symmetric MHD and VOD (44% and 73%, respectively). Both differences are statistically significant (MHD: χ^2^_(1)_ = 15.20, *p* < 0.001; VOD: χ^2^_(1)_ = 4.34, *p* = 0.037). The same is true for the rates at which defectors are punished efficiently, i.e. by *exactly one* group member. In line with H5, the rates of efficient punishment are higher in the asymmetric MHD and VOD (53% and 66%, respectively) than in the symmetric MHD and VOD (35% and 50%, respectively). Again, both differences are statistically significant (MHD: χ^2^_(1)_ = 18.96, *p* < 0.001; VOD: χ^2^_(1)_ = 12.93, *p* < 0.001).

These results show that punitive preferences and monetary incentives drive punishment decisions, but they also show that the efficiency at which punishment is carried out can be enhanced considerably through punishment cost heterogeneity. Punishment cost heterogeneity gives actors a simple means to tacitly coordinate on the optimal amount of punishment necessary to produce the second-order public good. The remaining question is whether (first-order) defection rates are indeed negatively correlated with the rates at which defectors are punished in the four experimental conditions.

**Figure 2:**
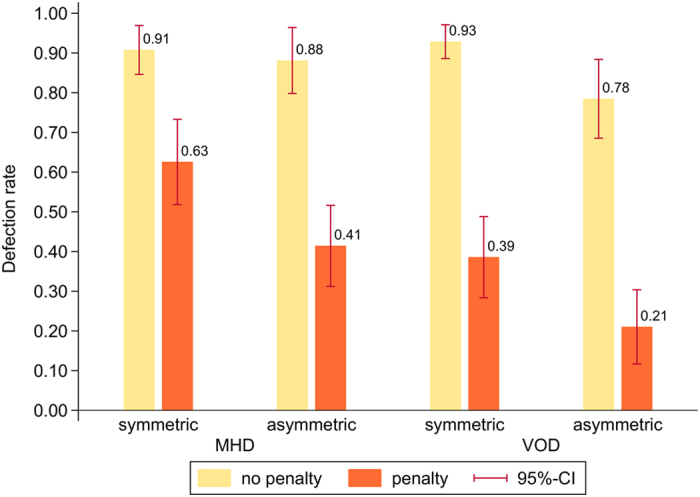
The figure shows defection rates across experimental conditions. The rates hardly differ without a penalty imposed on punished defectors. With a penalty, the rates drop significantly to levels that are inversely proportional to the punishment levels in the respective conditions. Except for the difference between the asymmetric MHD and the symmetric VOD, all differences between defection rates in the conditions with penalty are statistically significant. This shows that groups with an unequal distribution of punishment costs are more effective in deterring defections than groups of all equals. See section S2 in the *SI* for further details on the data analysis.

Figure 2 shows the defection (i.e., “stealing”) rates across experimental conditions. Unlike for punishment decisions, the penalty matters for first-order cooperation. Without a penalty imposed on punished defectors, defection rates remain very high and, except for the drop in the asymmetric VOD, barely differ across experimental conditions (χ^2^_(3)_ = 7.16, *p* = 0.067). As soon as a penalty is introduced in the second part of the experiment, defection rates fall drastically and reveal an interesting pattern. In line with hypothesis H6, defection rates are lowest in the asymmetric VOD (21%). Defection rates are highest in the symmetric MHD (63%), and they are intermediate in the asymmetric MHD and the symmetric VOD (41% and 39%, respectively). Although the difference between the last two conditions is statistically insignificant, the rank order of the defection rates in all four conditions is the inverse of the rank order of the rates at which defectors are punished (H6*e*).

## Discussion

The solution of the second-order public good problem is an important stepping stone to the solution of the first-order cooperation problem. It has been shown that decentralized peer-punishment can produce the second-order public good but often at a net cost for the group. These findings prompted scholars to invoke more organized forms of enforcement to explain cooperation, leaving the strategic nature of the punishment situation underexplored. Here we take a step back and show that dissecting the second-order public good problem can reveal some interesting and hitherto understudied mechanisms of second-order public good provision. We start from the assertion that in many situations only one group member is necessary and sufficient to produce the second-order public good. We thus model the second-order public good dilemma as a step-level public good game. This approach allows us to derive clear hypotheses that can be put to an empirical test.

The results of our laboratory experiment corroborate that punitive preferences and monetary incentives are important determinants of peer-punishment. More importantly, however, individual differences in punishment costs prove to be at least as effective in driving the punishment of defectors. First, punishment cost heterogeneity enables groups to tacitly coordinate on only the strongest group member to carry out the punishment thereby increasing the efficiency of second-order public good production. Second, with other things kept constant, groups in which punishment costs are unequally distributed are more effective in deterring defections than groups of all equals. These findings confirm that it can be fruitful to account for individual differences in evolutionary games^31^,^41^,^42^,^43^.

Future research should explore how other types of individual differences can promote coordinated action in the production of (second-order) public goods. Our study corroborates that punishment cost heterogeneity facilitates the tacit emergence of a designated punisher. However, the more general prediction, that individual differences in the net benefits from the second-order public good will produce the same results, has not yet been tested. Moreover, it would be interesting to see how groups of heterogeneous actors perform against groups of homogenous actors in which a designated punisher is randomly and explicitly appointed^22^.

In egalitarian societies, unequal endowments should not matter for individuals’ life-time outcomes. Inequality, however, may have arguably been an important element in the evolution of centralized punishment institutions. In a state of relative disorder, perceivable individual differences may have been a simple yet powerful means to coordinate action^44^. Our results show that even an arbitrary assignment of an individual to a focal position in the social hierarchy allows for the endogenous emergence of more centralized forms of punishment. Processes of cumulative advantage^45^,^46^, possibly paired with processes of territorial segregation^47^,^48^, may consolidate the power of those who happen to be stronger, and lead to new forms of organization which allow for cooperation in much larger groups than we are able to re-enact in our lab^31^.

Our findings help us understand how social order was possible in human prehistory, when centralized punishment institutions did not exist. It has been suggested that a possible next step in the transition from a state in which violence specialists maintain social order in small groups, to the next higher state of social organization, is the formation of dominant coalitions^28^. Members of the dominant coalition hold special functions (military, religious, political and economic) and privileges (material goods and power). By limiting access to these privileges, members of the coalition create incentives to cooperate rather than to fight with each other in the long run. Such cooperation thus requires that rents can be efficiently extracted and limited access to privileges continuously enforced. With regard to the latter, it appears more plausible to conjecture that a centralized punishment institution, such as pool punishment, would emerge to consolidate the violence potential of the coalition than a peer-punishment mechanism.

## Methods

### The volunteer’s dilemma and the missing hero dilemma

We start with the volunteer’s dilemma (VOD) to model the second-order public good problem^37^. The VOD is a step-level public good game where only one actor’s contribution is necessary and sufficient to produce the public good^15^. Here, punishing the thief to reclaim the stolen endowment for the entire group constitutes the public good. More formally (Table 2), a public good of value ∑*U*_*i*_ for a group of size *n* ≥ 2 is produced by a single actor *i* choosing C (punish) at a cost *K*_*i*_ where *U*_*i*_ > *K*_*i*_ > 0 " *i*. The public good is not provided if all actors choose D (not punish) and there is a welfare loss if more than one actor chooses C. We distinguish between a symmetric VOD, where *U*_*i*_ = *U*_*j*_ and *K*_*i*_ = *K*_*j*_ " *i* *≠ j*, and an asymmetr*i*c VOD^38^, where *U*_*i*_ ≠ *U*_*j*_ and/or *K*_*i*_ ≠ *K*_*j*_ $ *i* ≠ *j*.

Both the symmetric and asymmetric VOD have *n* Pareto-optimal Nash-equilibria in pure strategies, in which one group member chooses C and the *n – *1 other group members choose D. However, in the symmetric VOD, an equilibrium in pure strategies is not easily attainable without communication; although the benefits outweigh the costs of producing the public good, free riding on another group member’s punishment is even more beneficial. As a result, the entire group may end up losing part of their endowment to the thief while waiting for someone else to punish and reclaim the stolen amount. The symmetric VOD has a payoff-symmetric Nash-equilibrium in mixed strategies, which can be used to model this diffusion of responsibility effect^49^. How the mixed strategy equilibrium is derived can be seen elsewhere^15^,^37^,^38^. In the mixed strategy equilibrium (MSE), a group member *i*’s probability *p*_i_^*^ of choosing C is:
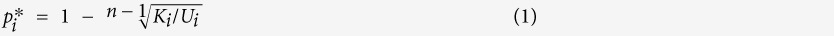
With *q*_i_^*^ = 1 – *p*_i_*, we can calculate the probability p^*^ that at least one group member will punish the defector and the second-order public good will be produced in the MSE:
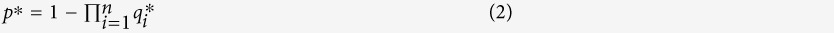
Note that both *p*_i_^*^ and p^*^ are decreasing in *n* (group size), decreasing in *K* (punishment cost) and increasing in *U* (size of the stolen endowment). In the asymmetric VOD, group member *i*’s probability *p*_i_^*^ of punishing in the MSE is:
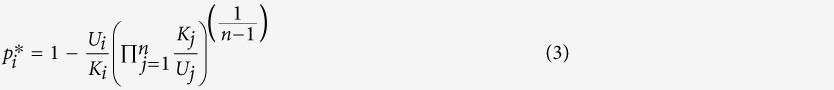


Unlike for the symmetric VOD, equation (3) implies that *p*_i_^*^ decreases as *U*_*i*_ increases and/or *K*_*i*_, decreases. In other words, the stronger a group member *i* is (in terms of benefits from and/or costs of producing the second-order public good) the lower is this group member’s probability to punish a defector. This is counter-intuitive and, moreover, for certain combinations of *U* and *K* an MSE does not exist. This makes the MSE not a very useful model of human behavior in the asymmetric VOD. However, alternative theoretical arguments^38^,^50^,^51^,^52^ as well as recent empirical evidence^32^ suggest that for the special case of an asymmetric VOD with one strongest group member, the pure strategy equilibrium will be selected in which the strongest group member chooses C and the rest of the group chooses D. In line with these theoretical arguments and empirical findings, a recent study has established that for the asymmetric VOD with one strong group member and *n* – 1 weak group members, the pure strategy equilibrium in which only the strong group member cooperates is evolutionary stable^29^.

The volunteer’s dilemma turns into a missing hero dilemma (MHD) if the punishment costs exceed the benefits^36^, that is, if *K*_*i*_ > *U*_*i*_ > 0 " *i*. Unlike in the VOD, choosing D is a dominant strategy in the MHD and there is a unique Nash-equilibrium (in pure strategies), in which all group members choose D, irrespective of whether the game is symmetric or asymmetric.

### Model predictions and hypotheses

Based on the payoff structures of the stage games specified in Table 1, the probabilities of second-order public good provision (i.e. punishment) at the individual (*p*_i_^*^) and the group level (p^*^) can be calculated from equations (1) and (2), respectively (see upper half of Table 3). Clearly, in the MHD, the punishment probabilities will be zero both in the symmetric and the asymmetric versions of the game. Plugging the numbers for the symmetric VOD into equations (1) and (2) yields *p*_i_^*^ = 0.293 and p^*^ = 0.646, respectively. As mentioned above, in the asymmetric VOD, we base our expectations on an alternative theoretical model^29^,^50^,^51^,^52^. For the asymmetric VOD, we expect that only the strongest group member (*i* = 2) will carry out the punishment (*p*_2_^*^ = 1 and *p*_1_^*^ = *p*_3_^*^ = 0) and thus the second-order public good will always be produced (p^*^ = 1). The latter also implies that the punishment will always be carried out by one person only and, therefore, the second-order public good will always be produced efficiently. In the symmetric VOD, the probability that punishment will be carried out by one person only is 0.439 [*np*_i_^*^(1 – *p*_i_^*^)^*n*-1^].

Recall that in each round of our experiment subjects are randomly assigned to be the potential thief (i.e. defector) or one of the other three group members. Subjects could therefore adopt the “always steal and never punish” strategy. This strategy would produce both efficient and equal outcomes. However, “never punish” is not an equilibrium strategy in the symmetric VOD, and it is not an equilibrium strategy for all group members in the asymmetric VOD. Moreover, since groups are randomly formed anew after each round, it is hardly possible to enforce a “never punish” strategy by means of trigger strategies, for instance. Thus, in accordance with the above predictions, some of the subjects always have an incentive to punish and reclaim their stolen endowments in the VOD, but none ever do in the MHD.

Based on our model predictions, and the assumption that actors are rational and self-regarding, we can state our hypotheses with regard to punishment:

**H1**: Punishment rates will be zero in both the symmetric and asymmetric MHD.

**H2**: Punishment rates will be higher in the VOD than in the MHD.

**H3**: In the asymmetric VOD, the strong group member will more often punish defectors than a weak group member.

**H4**: In the asymmetric VOD, defectors will be punished more often than in the symmetric VOD.

**H5**: In the asymmetric VOD, punishment will be carried out more efficiently (i.e. more often by one group member only) than in the symmetric VOD.

Based on the predicted probabilities that punishment will be carried out by at least one group member (p^*^), a potential thief’s expected gain from stealing [π_X_(s)] can be calculated both for the condition without and the condition with an extra penalty (see the bottom half of Table 3). Note first that the gains from not stealing [π_X_(¬s)] are always zero. Hence, whenever the gains from stealing are larger than zero, we can expect actors to steal with certainty. For example, in the symmetric VOD, a thief’s expected gain from stealing is 150 MU × (1 – 0.646) = 53.1 MU if there is no penalty, and 150 MU × (1 – 0.646) – 60 MU × 0.646 = 14.3 MU if there is a penalty. Thus, our hypothesis regarding (first-order) defection can be stated as follows:

**H6**: Defection rates will be lower in the asymmetric VOD than in the symmetric VOD and the MHD.

If we now assume also other-regarding actors with punitive preferences, we can state an alternative hypothesis to H1:

**H1*****a***: Punishment rates will be larger than zero in both the symmetric and asymmetric MHD.

Models of other-regarding preferences usually imply that actors attach larger weights to own monetary gains and losses than to others’ monetary gains and losses^53^. Thus, assuming also other-regarding actors with punitive preferences does not change hypothesis H2 of higher punishment rates in the VOD conditions than in the MHD conditions. Moreover, hypotheses H3 through H5 can now also be tested with the MHD. That is, we can expect that in the asymmetric MHD the strong group member will punish defectors more often than a weak group member (H3); that in the asymmetric MHD defectors will be punished more often than in the symmetric MHD (H4); and that in the asymmetric MHD punishment will be carried out more efficiently than in the symmetric MHD (H5). We can extend hypothesis H6 to reflect the empirical punishment rates. Correspondingly, (first-order) defection rates should be negatively correlated with the rates at which defectors are punished. In particular, defection will be lower in the asymmetric VOD than in the symmetric MHD:

**H6*****e***: Defection rates in the four experimental conditions will be inversely proportional to the rates at which defectors are punished in the four conditions.

Originally, we also expected that those who steal in the penalty condition (i.e. second part of the experiment) will be perceived as more provocative, and may therefore induce more emotion-driven and spiteful punishment. Consequently, we expected to observe higher punishment rates across all conditions in the second part of the experiment. In fact, in all experimental conditions are punishment rates higher in the second part than in the first part, both at the individual and the group level, and the difference is statistically significant in the symmetric VOD condition (see Figure S5 in the *SI*). However, testing this hypothesis was not central to our paper. We therefore decided not to expand on this result in the main part of the paper.

### Experimental procedure

In total, 216 subjects participated in our computerized laboratory experiment. The experiment comprised six sessions and 36 subjects participated in each session. Subjects were students from the University of Zurich and ETH Zurich, 57.9% were female and they were 23.1 years old on average (sd = 5.57). Upon arrival in the lab, subjects were randomly assigned to two of the four experimental conditions. Table 4 shows the sequence in which the experimental conditions were tested. Subjects received condition-specific instructions on paper. The instructions that were given to subjects in one of the experimental conditions (asymmetric MHD) are reproduced in figures S1 through S4 in the *SI* (translated from German by the authors). Instructions explained the decision situations step by step and contained shots of the actual decision screens. Moreover, subjects learned that their decisions were anonymous, that their payments would correspond to the sum they earned in each round and that payments would be administered by a person not involved in the implementation of the experiment. After reading the instructions, subjects took a quiz with questions about the decision situations. Questions for which at least one wrong answer was given were read out loud and the correct answer was explained to all subjects at the same time. Then, the experiment started. A session lasted for about 1h and subjects earned CHF 38 (incl. CHF 10 show-up fee) on average (≈USD 43.4). After the experiment, subjects filled in a questionnaire and could leave the lab to get their payment in private. The experiment was programmed and conducted with the software z-Tree^54^.

### Statement of research conduct

All the research was performed in the Decision Science Laboratory (DeSciL) at ETH Zurich, Haldeneggsteig 4, CH-8092 Zurich, Switzerland. The review board of DeSciL is called DeSciL Review Board, and its members are listed on the DeSciL website (https://www.descil.ethz.ch/people). Our experiment was conducted in accordance with DeSciL Operational Rules, which are approved by the review board and published on the DeSciL website (https://www.descil.ethz.ch/research/policies). All participants in our experiment were recruited from the subject pool maintained by the University Registration Center for Study Participants (UAST) of the University of Zurich and ETH Zurich. Every person who has signed up to this subject pool also gave his or her informed consent by agreeing to the terms and conditions of UAST. These terms and conditions are published on the UAST website (https://www.uast.uzh.ch/register).

## Author Contributions

A.D. and W.P. conceptualized and designed the experiment; W.P. implemented and conducted the experiment, and analyzed the data; A.D. and W.P. wrote the paper.

## Additional Information

**How to cite this article**: Diekmann, A. and Przepiorka, W. Punitive preferences, monetary incentives and tacit coordination in the punishment of defectors promote cooperation in humans. *Sci. Rep.* **5**, 10321; doi: 10.1038/srep10321 (2015).

## Supplementary Material

Supplementary Information

## Figures and Tables

**Table 1:** Experimental games and design.

	**MHD**		**VOD**	
	**symmetric** ***U***_***i***_ **=** **50** **MU** ***K***_***i***_ **=** **55** **MU**	**asymmetric*****U***_***i***_ **=** **50** **MU*****K***_**1,3**_ **=** **65** **MU*****K***_**2**_ **=** **55** **MU**	**symmetric*****U***_***i***_ **=** **50** **MU*****K***_***i***_ **=** **25** **MU**	**asymmetric*****U***_***i***_ **=** **50** **MU*****K***_**1,3**_ **=** **35** **MU*****K***_**2**_ **=** **25** **MU**
part 1:	no penalty	no penalty	no penalty	no penalty
rounds 1–15	*P* **=** 0 **MU**	*P* **=** 0 **MU**	*P* **=** 0 **MU**	*P* **=** 0 **MU**
part 2:	penalty	penalty	penalty	penalty
rounds 16–30	*P* **=** 60 **MU**	*P* **=** 60 **MU**	*P* **=** 60 **MU**	*P* **=** 60 **MU**

The table shows the varying structure of the second-order public good game across experimental conditions. *U*_*i*_ denotes the stolen endowment a group member can reclaim; *K*_*i*_ denotes the costs a group member incurs if they decide to reclaim the stolen endowment; *P* denotes the penalty a thief incurs if the stolen endowment is reclaimed by at least one group member. MU stands for monetary units; 100 MU correspond CHF 1 (≈USD 1.14). In the experiment, we varied the structure of the second-order public good game between-subject, and whether or not a punished defector incurred an extra penalty within-subject. Six sessions were conducted with 36 participants in each session (*N* = 216). In each session, participants were randomly assigned to two of the four experimental conditions. Participants interacted in groups of four which were randomly formed anew in each round. Before a group was disbanded, all group members received full information feedback about the outcome of their interaction and learned how every group member had decided. See the Methods section for further details on the experimental design.

**Table 2:** The volunteer’s dilemma (VOD).

	**Number of other persons choosing C**			
Person *i*’s choice	0	1	…	*n* - 1
C: punish	*U*_*i*_ *- K*_*i*_	*U*_*i*_ *- K*_*i*_	*U*_*i*_ *- K*_*i*_	*U*_*i*_ *- K*_*i*_
D: not punish	0	*U*_*i*_	*U*_*i*_	*U*_*i*_

**Table 3:** Predicted punishment probabilities and thief’s incentives to steal.

	**Predicted punishment probabilities**										
	**MHD**				**VOD**						
	**symmetric**		**asymmetric**		**symmetric**		**asymmetric**				
*p*_1,3_^*^	0		0		0.293		0	
*p*_2_^*^	0		0		0.293		1	
p^*^	0		0		0.646		1	
	symmetric		asymmetric		symmetric		asymmetric	
Penalty	no	yes	no	yes	no	yes	no	yes
π_X_(s)	150	150	150	150	53.1	14.3	0	−60
π_X_(¬s)	0	0	0	0	0	0	0	0

**Table 4:** Experimental conditions tested per session.

**Session**	**Condition 1 (subjects 1–16)**	**Condition 2 (subjects 17–36)**
1	asym. VOD	asym. MHD
2	sym. VOD	sym. MHD
3	sym. MHD	asym. VOD
4	asym. MHD	sym. VOD
5	sym. MHD	asym. MHD
6	asym. VOD	sym. VOD
