# Supplementary material for: Punitive preferences, monetary incentives and tacit coordination in the punishment of defectors promote cooperation in humans
Source: Sci Rep. 2015 May 19;5:10321. doi: 10.1038/srep10321 (PMC4437292; doi:10.1038/srep10321)
Supplement: Supplementary Information [file srep10321-s1.pdf]

Supplementary Information (*SI*) for:

Punitive preferences, monetary incentives and tacit coordination in the punishment of defectors promote cooperation in humans

Andreas Diekmann[1] and Wojtek Przepiorka[2,3,*]

[1] Chair of Sociology, ETH Zurich, Clausiusstrasse 50, CLU D 3, CH-8092 Zurich, Switzerland

[2] Department of Sociology, University of Oxford, Manor Road, Oxford, OX1 3UQ, United Kingdom

[3] Department of Sociology, Utrecht University, Padualaan 14, Utrecht, 3584 CH, The Netherlands

[*] To whom correspondence should be addressed. Phone: +31-30-253-8814; E-Mail: w.przepiorka@uu.nl

S1. Experimental instructions

Introduction

Please read these instructions attentively.

The experiment will last for about 60 minutes, it consists of two parts and each part comprises 15 rounds. You are receiving the instructions concerning the first part of the experiment on these three pages, which you may use as a reminder during the experiment. Instructions concerning the second part are short and you will receive them at the end of the first part on your screen only. Let us start with some important information.

You are participating in an experiment in which you will earn some money. The amounts you earn in each of the 30 rounds will be summed up at the end, and you will be paid this amount together with your show-up fee of 10 sFr. in cash. The payment will be made by an assistant that was not involved in the implementation of this experiment. Your earnings depend on both the decisions you will make and the decisions other participants will make. There are no right or wrong decisions and you make all your decisions anonymously.

All participants are receiving the same instructions and take part in this experiment under the same conditions. Please, from now on, do not talk to each other anymore and switch off your mobile devices. This experiment is being conducted by the Chair of Sociology at ETH Zurich and the data collected in this experiment are for scientific purposes only.

1

Figure S1: Instructions (asymmetric MHD; page 1)

Instructions (Part 1)

The first part of the experiment will last for 15 rounds. In each round you are with three different people in a group and a group consists of a *Person 1*, a *Person 2*, a *Person 3* and a *Person X*. That is, at the beginning of each round, the groups are formed randomly anew (that is, in each round you will be with different participants in a group) and it is determined by chance whether you are *Person 1*, *Person 2*, *Person 3* or *Person X*.

In each round, each participant receives an endowment of 100 Rp. (= 1 sFr.). *Person X* (and only *Person X*) can then decide whether or not he or she would like to deduct part of the other three persons' endowments (see Figure 1). *Person X* can deduct either 0 Rp. or 50 Rp. from each of the other persons' endowments (i.e. 0 Rp. or 3 x 50 Rp. = 150 Rp.) and this amount will be added to *Person X*'s account. That is, if *Person X* decides to deduct 50 Rp. from each of the other three persons, then *Person X*'s account will amount to 250 Rp. (100 Rp. + 150 Rp. = 250 Rp.) and the other three persons' accounts will amount to 50 Rp. each (100 Rp. – 50 Rp. = 50 Rp.). If *Person X* decides to deduct 0 Rp. from the other three persons, then all four persons will keep their endowment of 100 Rp.

Figure 1: Decision of Person X

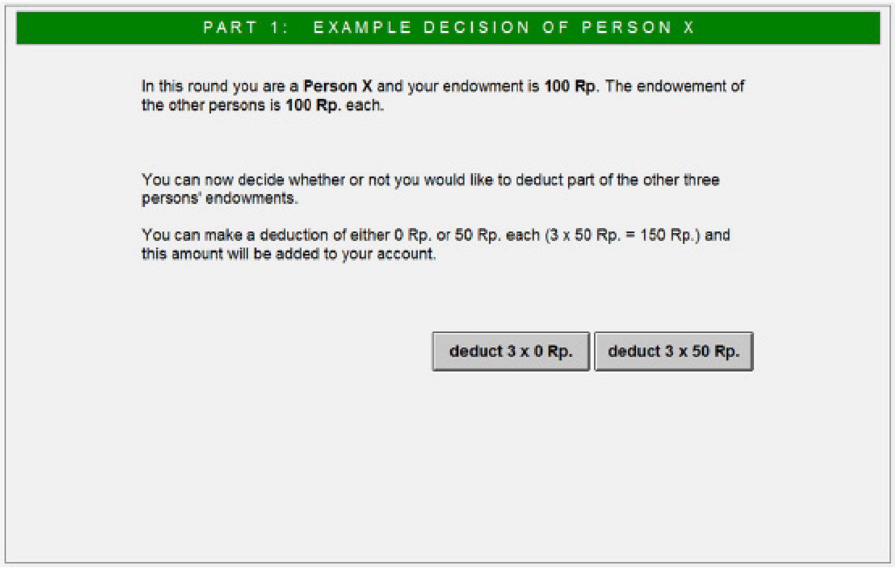

2

Figure S2: Instructions (asymmetric MHD; page 2)

If *Person X* decides to deduct 50 Rp. from each of the other three persons, then these three persons can decide independently whether or not they want to reclaim this amount from *Person X*. If *Person X* decides not to deduct anything from the other three persons, then, of course, these three persons do not have the possibility to reclaim anything from *Person X*.

Hence, if *Person X* decides to deduct 50 Rp. from each of the other three persons, the decision situation depicted in Figure 2 appears on the other three persons' screens. Then, the three persons decide independently whether they want to choose "up" or "down" in their decision field. If at least one of the three chooses "up", the account of *Person X* will be reduced back to 100 Rp. (250 Rp. – 150 Rp. = 100 Rp.) and the other three persons' accounts will amount to 100 Rp. each again (50 Rp. + 50 Rp. = 100 Rp.). However, *Person 2* will be charged 55 Rp. if he or she chooses "up". *Person 1* will be charged 65 Rp. for choosing "up" and *Person 3* will also be charged 65 Rp. for choosing "up". A person that chooses "down" will not be charged anything. If, however, all three persons choose "down", the amount that *Person X* deducted from their accounts will not be reclaimed.

Figure 2: Decision of Person 1, 2, and 3

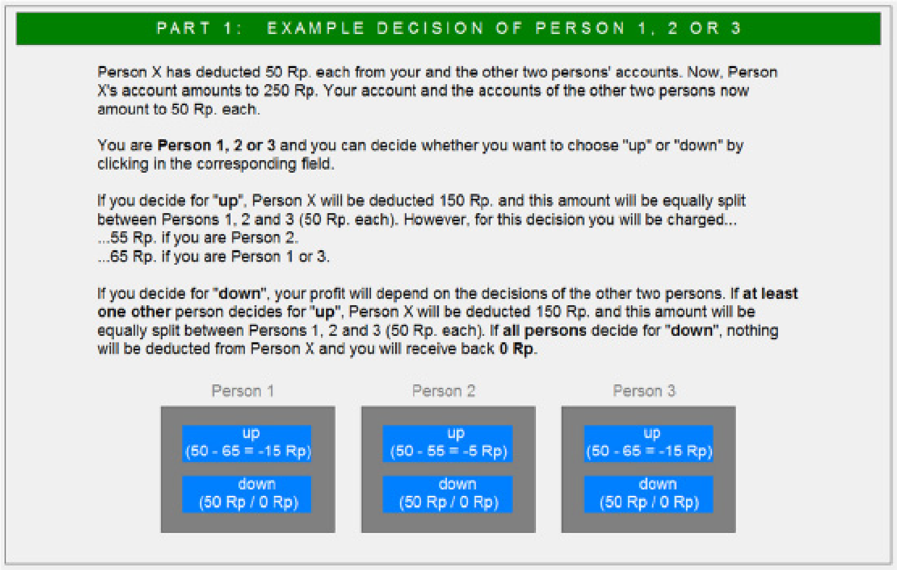

Before the next round, the groups of four will be disbanded and formed randomly anew, and it will be determined by chance whether you are *Person 1*, *Person 2*, *Person 3* or *Person X*. It could therefore be that you will never be Person X, for instance, or only at irregular intervals.

3

Figure S3: Instructions (asymmetric MHD; page 3)

Instructions (Part 2) [on screen only, after Part 1]

The second part of the experiment will last for 15 rounds and differs from the first part of the experiment as follows:

If *Person X* decides to deduct 50 Rp. from each of the other three persons and then at least one of the other three persons chooses “up”, the account of *Person X* will be reduced by an additional 60 Rp. to 40 Rp. instead of 100 Rp. as before. *Person X*'s account will always be reduced at most by an additional 60 Rp., irrespective of whether one, two or all three of the other persons choose “up”. If all three persons choose “down”, the amount that *Person X* deducted from their accounts will not be reclaimed and the account of *Person X* will not be additionally reduced. Everything else stays the same.

4

Figure S4: Instructions (asymmetric MHD; page 4)

S2. Further data analyses and results

All test statistics reported in the main article and figures 1 and 2 are based on the regression model estimations presented in this section. Statistical significance is set at the 5% level (i.e. $\alpha = 0.05$) for two-sided tests and we account for the repeated measures obtained on the same subject by estimating *cluster*-robust standard errors. We use Stata's *margins* command to calculate proportions from *logit* regressions and test the statistical significance of the differences between proportions using Wald tests of linear hypotheses. Regression tables are created using the *estout* command in Stata[S1]. The data are available from the authors on request.

Table S1 lists the coefficient estimates from logit regression models of individual group members' punishment decisions. Model M1 accounts for the type of the stage game only (MHD vs. VOD). The overall punishment rates in the MHD (19.8 %) and in the VOD (32.7 %) are significantly different from each other ($\chi^2_{(1)} = 15.61, p < 0.001$). Model M2 shows that the difference in punishment rates between the MHD and the VOD is also statistically significant within both the symmetric (17.7 % vs. 32.9 %; $\chi^2_{(1)} = 9.04$, $p = 0.003$) and the asymmetric (22.2 % vs. 32.6 %; $\chi^2_{(1)} = 9.30$, $p = 0.002$) versions of the games. Model M3 accounts for the entire structure of the stage games, but it does not differentiate between the first part (without a penalty) and the second part of the experiment (with a penalty). This model shows that a weak person is much less likely to punish defectors than the strong person in both the asymmetric MHD (5.1 % vs. 56.3 %; $\chi^2_{(1)} = 81.99, p < 0.001$) and the asymmetric VOD (10.6 % vs. 76.8 %; $\chi^2_{(1)} = 119.44, p < 0.001$).

Model M4 is the most unrestricted model as it also accounts for the two parts of the experiment. The punishment rates displayed in Figure 1 are based on this model. Except for the difference in the symmetric VOD ($\chi^2_{(1)} = 6.50$, $p = 0.011$), the differences between the punishment rates in the first part (without penalty) and the second part of the experiment (with penalty) are all statistically insignificant. Based on this result, and the fact that the punishment rates do not increase substantially from the first to the second part, we feel confident that reporting results regarding the punishment of defectors based on the pooled data from both parts does not obfuscate any relevant facts.

Table S1: Regression models of punishment at individual level

	M1		M2		M3		M4	
	Coef.	SE	Coef.	SE	Coef.	SE	Coef.	SE
MHD	-1.396***	0.103						
× symmetric			-1.535***	0.159	-1.535***	0.159		
× no penalty							-1.672***	0.183
× penalty							-1.356***	0.196
× asymmetric			-1.255***	0.130				
× weak person					-2.914***	0.278		
× no penalty							-3.164***	0.324
× penalty							-2.516***	0.435
× strong person					0.251	0.231		
× no penalty							0.141	0.245
× penalty							0.492	0.325
VOD	-0.719***	0.129						
× symmetric			-0.715***	0.202	-0.715***	0.202		
× no penalty							-0.835***	0.194
× penalty							-0.443	0.250
× asymmetric			-0.724***	0.118				
× weak person					-2.136***	0.297		
× no penalty							-2.147***	0.297
× penalty							-2.093***	0.428
× strong person					1.197***	0.283		
× no penalty							1.107***	0.292
× penalty							1.580***	0.419
N_1	3123		3123		3123		3123	
N_2	216		216		216		216	
pseudo R^2	0.019		0.021		0.170		0.175	
$\chi^2(df)$	16.94(1)***		22.18(3)***		171.26(5)***		183.17(11)***	

Notes: The table lists coefficient estimates from logistic regression models and cluster-robust standard errors (*** $p < 0.001$, ** $p < 0.01$, * $p < 0.05$, for two-sided tests). The models were estimated without a constant so that the coefficients can be interpreted as deviations from zero (i.e., 0.5). Goodness of fit measures are based on model estimations with a constant. The outcome variable in all models is 1 if a person punished the defection and is 0 otherwise. Figure 1 is based on the estimates in model M4. N_1 denotes the number of decisions and N_2 denotes the number of clusters.

Table S2 lists the coefficient estimates from logit regression models of punishment at the group level. In these models, the outcome variable is 1 if the defection was punished by *at least one* group member, and it is 0 otherwise. Here too, model M5 only accounts for the type of the stage game (MHD vs. VOD). Defectors are punished at a significantly lower overall rate in the MHD than in the VOD (51.5 % vs. 76.2 %; $\chi^2_{(1)} = 73.89$, $p < 0.001$). Model M6 confirms that this also holds within the symmetric (43.8 % vs. 72.8 %; $\chi^2_{(1)} = 54.60$, $p < 0.001$) and the asymmetric (59.9 % vs. 80.9 %; $\chi^2_{(1)} = 26.25$, $p < 0.001$) versions of the games. As stated in the main paper, there is also a significant difference in punishment rates between

the symmetric and asymmetric MHD (44 % vs. 60 %; $\chi^2_{(1)} = 15.20$, $p < 0.001$) and the symmetric and asymmetric VOD (73 % vs. 81 %; $\chi^2_{(1)} = 4.34$, $p = 0.037$).

Table S2: Regression models of punishment at group level

	M5		M6		M7	
	Coef.	SE	Coef.	SE	Coef.	SE
MHD	0.060	0.084				
× symmetric			-0.249*	0.117		
× no penalty					-0.377*	0.153
× penalty					-0.066	0.181
× asymmetric			0.402**	0.124		
× no penalty					0.250	0.148
× penalty					0.745**	0.230
VOD	1.162***	0.108				
× symmetric			0.986***	0.135		
× no penalty					0.811***	0.155
× penalty					1.482***	0.286
× asymmetric			1.445***	0.183		
× no penalty					1.330***	0.199
× penalty					1.974***	0.477
N	1041		1041		1041	
pseudo R^2	0.050		0.064		0.072	
$\chi^2(df)$	64.82(1)***		81.26(3)***		88.92(7)***	

Notes: The table lists coefficient estimates from logistic regression models and heteroscedasticity-robust standard errors (*** $p < 0.001$, ** $p < 0.01$, * $p < 0.05$, for two-sided tests). The models were estimated without a constant so that the coefficients can be interpreted as deviations from zero (i.e., 0.5). Goodness of fit measures are based on model estimations with a constant. The outcome variable in all models is 1 if the defection was punished by at least one group member and is 0 otherwise. Figure S5 is based on the estimates in model M7.

Recall that the latter two differences are estimated based on the pooled data from both parts of the experiment. Model M7 also accounts for the two parts of the experiment and allows for testing the differences between the symmetric and asymmetric games for each part separately. Except for the difference in the VOD with penalty ($\chi^2_{(1)} = 0.89$, $p = 0.345$), the differences in punishment rates between the symmetric and asymmetric games are all statistically significant. Note, however, that the insignificant difference in the VOD with penalty may partly be due to low power in the data; defection rates are lowest in the VOD with penalty (see Fig. 2) making it most difficult to identify a statistically significant difference in this experimental condition. We are therefore confident that reporting results regarding the punishment rates at the group level based on the pooled data from both parts does not

obfuscate any relevant facts. The punishment rates displayed in Figure S5 are based on model M7.

Figure S5: Punishment rate at group level

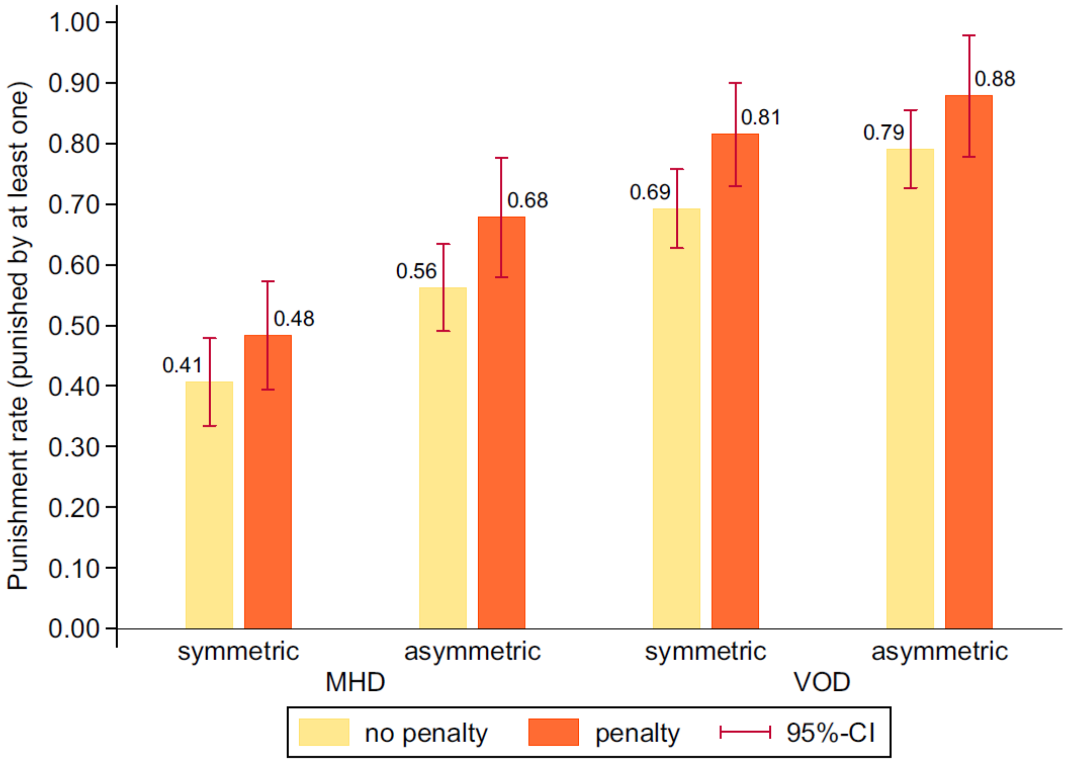

Table S3 lists the coefficient estimates from logit regression models of single group member punishment. The outcome variable in all three models is 1 if the defection was punished by *exactly one* group member, and it is 0 otherwise. In other words, these models estimate the rates at which the second-order public good is produced efficiently. Again, model M8 distinguishes between the two types of the stage game only (MHD vs. VOD). The second-order public good is produced less often efficiently in the MHD than in the VOD, both overall (44.0 % vs. 56.4 %; $\chi^2_{(1)} = 16.16$, $p < 0.001$) as well as within the symmetric (35.5 % vs. 49.6 %; $\chi^2_{(1)} = 12.03$, $p = 0.001$) and the asymmetric (53.3 % vs. 66.0 %; $\chi^2_{(1)} = 12.03$, $p = 0.001$) versions of the games (based on model M9). However, what is of greater substantial importance here is the difference between the symmetric and asymmetric versions of the games. These differences can also be estimated based on model M9. As mentioned in the main paper, the rate of efficient public good provision is significantly lower in the symmetric MHD than in the asymmetric MHD (35.5 % vs. 53.3 %; $\chi^2_{(1)} = 18.96$, $p < 0.001$), and it is

significantly lower in the symmetric VOD than in the asymmetric VOD (49.6 % vs. 66.0 %; $\chi^2_{(1)} = 12.93$, $p < 0.001$).

Model M10 is the most unrestricted model as it also accounts for the two parts of the experiment. The rates of efficient public good provision displayed in Figure S6 are based on this model. None of the differences in single punisher rates between the first part (without penalty) and the second part of the experiment (with penalty) are statistically significant.

Table S3: Regression models of punishment by a single group member (group level)

	M8		M9		M10	
	Coef.	SE	Coef.	SE	Coef.	SE
MHD	-0.243**	0.084				
× symmetric			-0.599***	0.121		
× no penalty					-0.643***	0.158
× penalty					-0.537**	0.188
× asymmetric			0.133	0.122		
× no penalty					0.032	0.147
× penalty					0.348	0.218
VOD	0.257**	0.093				
× symmetric			-0.014	0.120		
× no penalty					-0.051	0.143
× penalty					0.074	0.222
× asymmetric			0.662***	0.152		
× no penalty					0.578***	0.169
× penalty					1.003**	0.353
N	1041		1041		1041	
pseudo R^2	0.011		0.033		0.035	
$\chi^2(df)$	15.82(1)***		44.77(3)***		47.28(7)***	

Notes: The table lists coefficient estimates from logistic regression models and heteroskedasticity-robust standard errors (*** $p < 0.001$, ** $p < 0.01$, * $p < 0.05$, for two-sided tests). The models were estimated without a constant so that the coefficients can be interpreted as deviations from zero (i.e., 0.5). Goodness of fit measures are based on model estimations with a constant. The outcome variable in all models is 1 if the defection was punished by exactly one group member and is 0 otherwise. Figure S6 is based on the estimates in model M10.

Figure S6: Single punisher rate

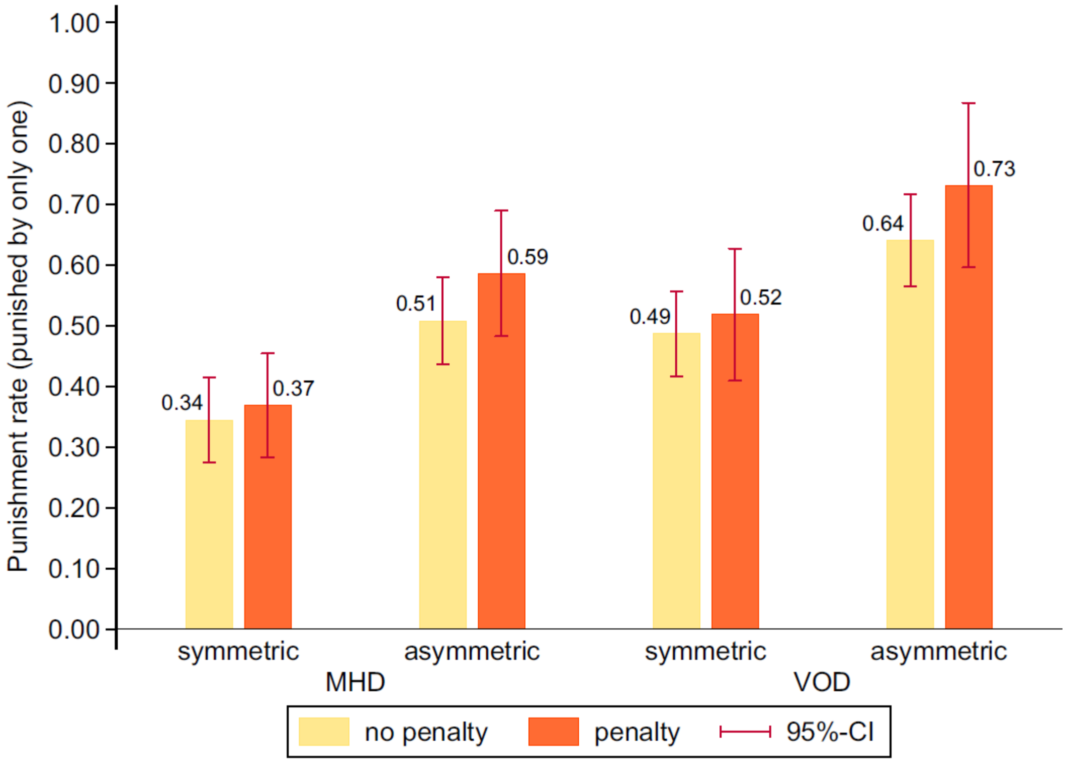

Finally, Table S4 lists the coefficient estimates from logit regression models of defections. The outcome variable in all three models is 1 if a defection occurred, and it is 0 otherwise. Based on models M11 and M12, we can see that defection rates are higher in the MHD than in the VOD overall (70.5 % vs. 58.0 %; $\chi^2_{(1)} = 10.34$, $p = 0.001$), as well as within the symmetric (76.7 % vs. 65.7 %; $\chi^2_{(1)} = 5.04$, $p = 0.025$) and the asymmetric (64.8 % vs. 49.7 %; $\chi^2_{(1)} = 6.77$, $p = 0.009$) versions of the games, respectively. Again, what may be of more substantial importance here is the difference in defection rates between the symmetric and asymmetric versions of the games. These differences can also be estimated based on model M12. The defection rate is significantly higher in the symmetric MHD than in the asymmetric MHD (76.7 % vs. 64.8 %; $\chi^2_{(1)} = 4.77$, $p = 0.029$), and it is significantly higher in the symmetric VOD than in the asymmetric VOD (65.7 % vs. 49.7 %; $\chi^2_{(1)} = 9.31$, $p = 0.002$).

Unlike in the case of punishment rates, the latter two differences are somewhat obfuscated by the fact that model M12 does not distinguish between the first part (without penalty) and the second part of the experiment (with penalty). As mentioned in the main paper, the penalty matters a great deal with regard to defection. Model M13 shows that in the first part of the experiment (without a penalty), only the difference between the symmetric and asymmetric VOD is statistically significant ($\chi^2_{(1)} = 6.84$, $p = 0.009$). As soon as a penalty is introduced in

the second part, overall defection rates drop dramatically (87.7 % vs. 40.9 %; $\chi^2_{(1)} = 252.23$, $p < 0.001$). From model M13 we can see that defection rates in the second part depend substantially on the experimental condition. Defection rates are higher in the symmetric MHD than in the asymmetric VOD (62.6 % vs. 21.0 %; $\chi^2_{(1)} = 32.77$, $p < 0.001$); they are higher in the symmetric MHD than in the asymmetric MHD (62.6 % vs. 41.4 %; $\chi^2_{(1)} = 7.83$, $p = 0.005$); and they are higher in the symmetric VOD than in the asymmetric VOD (38.6 % vs. 21.0 %; $\chi^2_{(1)} = 6.17$, $p = 0.013$). The difference in defection rates between the asymmetric MHD and the symmetric VOD is statistically insignificant (41.4 % vs. 38.6 %; $\chi^2_{(1)} = 0.15$, $p = 0.698$). The defection rates displayed in Figure 2 are based on model M13.

Table S4: Regression models of defection

	M11		M12		M13	
	Coef.	SE	Coef.	SE	Coef.	SE
MHD	0.871***	0.134				
× symmetric			1.190***	0.209		
× no penalty					2.286***	0.374
× penalty					0.514*	0.234
× asymmetric			0.609***	0.174		
× no penalty					2.001***	0.404
× penalty					-0.346	0.215
VOD	0.324**	0.111				
× symmetric			0.651***	0.139		
× no penalty					2.565***	0.328
× penalty					-0.465*	0.220
× asymmetric			-0.010	0.168		
× no penalty					1.293***	0.299
× penalty					-1.323***	0.287
N_1	1620		1620		1620	
N_2	216		216		216	
pseudo R^2	0.013		0.030		0.239	
$\chi^2(df)$	9.91(1)**		21.07(3)***		188.56(7)***	

Notes: The table lists coefficient estimates from logistic regression models and cluster-robust standard errors (*** $p < 0.001$, ** $p < 0.01$, * $p < 0.05$, for two-sided tests). The models were estimated without a constant so that the coefficients can be interpreted as deviations from zero (i.e., 0.5). Goodness of fit measures are based on model estimations with a constant. The outcome variable in all models is 1 if a defection occurred and is 0 otherwise. Figure 2 is based on the estimates in model M13.

References

S1. Jann, B. Making regression tables simplified. *Stata J.* **7,** 227–244 (2007).
